# Solid-diffusion-facilitated cleaning of copper foil improves the quality of CVD graphene

**DOI:** 10.1038/s41598-018-36390-4

**Published:** 2019-01-22

**Authors:** Dinh-Tuan Nguyen, Wan-Yu Chiang, Yen-Hsun Su, Mario Hofmann, Ya-Ping Hsieh

**Affiliations:** 1 Department of Materials Science and Engineering, National Cheng Kung University, Tainan, 70101 Taiwan; 2 Graduate Institute of Opto-Mechatronics, National Chung Cheng University, Chiayi, 62102 Taiwan; 3 Department of Physics, National Taiwan University, Taipei, 10617 Taiwan; 4 Institute for Atomic and Molecular Science, Academia Sinica, Taipei, 10617 Taiwan

## Abstract

The quality of CVD-grown graphene is limited by the parallel nucleation of grains from surface impurities which leads to increased grain boundary densities. Currently employed cleaning methods cannot completely remove surface impurities since impurity diffusion from the bulk to the surface occurs during growth. We here introduce a new method to remove impurities not only on the surface but also from the bulk. By employing a solid cap during annealing that acts as a sink for impurities and leads to an enhancement of copper purity throughout the catalyst thickness. The high efficiency of the solid-diffusion-based transport pathway results in a drastic decrease in the surface particle concentration in a relatively short time, as evident in AFM and SIMS characterization of copper foils. Graphene grown on those substrates displays enhanced grain sizes and room-temperature, large-area carrier mobilities in excess of 5000 cm^2^/Vs which emphasizes the suitability of our approach for future graphene applications.

## Introduction

Graphene is a two-dimensional carbon material that could revolutionize high-speed electronics and sensors^1,2^. Chemical vapor deposition (CVD) on copper foil stands as the most promising method for large-scale production of high quality, single layer graphene. Much effort has been invested on bringing CVD graphene quality to the level of pristine mechanically exfoliated sample^3^. The largest hurdle to this goal is the polycrystallinity of CVD graphene, for the grain boundaries are the main factor of electron scattering in graphene lattice, which affects carrier mobility^4^.

To limit the parallel growth of grains, the nucleation process has to be controlled. Several reports emphasize the role of surface impurities on the copper substrate in the nucleation of graphene^5–7^. It has been suggested that their curvature results in local supersaturation of carbon in the growth substrate and subsequent precipitation into a graphene seed^8,9^, and some impurities have lower nucleation barrier than Cu and serve as heteronuclei in early growth^10^.

Therefore, substantial work has been done to clean and smoothen copper substrate, ranging from annealing, wet etching to mechanical and electrochemical polishing^3,5,11–15^ with varying degrees of success. While such strategies can remove surface contamination, they cannot remove impurities in the bulk substrate that will precipitate to the surface throughout the growth process. Annealing can improve copper morphology due to the evaporation of impurities. However, the presence of impurities even after 3 hours of annealing^6^ suggests that the evaporation process is too slow to deplete the impurities close to the surface before new ones can diffuse from the bulk. Moreover, impurities can form refractory oxides or alloy with Cu, which are immune to evaporative removal.

We here introduce a solid-diffusion-based purification process of the copper substrate to overcome these issues. Copper is brought in intimate contact with a solid cap, which allows inter-diffusion at elevated temperatures. Due to the high atomic mass of Cu, it diffuses slower than most of its impurities which results in a purification of the growth substrate.

We show that the purification process results in a significant change in the impurity distribution within the copper foil. The diffusion process was found to control the particle density on the copper foil surface and ultralow particle densities could be obtained. Our model of solid diffusion was validated by a comparison of different cap materials. Graphene grown on these copper foils exhibited enhanced quality as confirmed by spectroscopic and carrier-transport measurements.

## Results and Discussion

**Figure 1:**
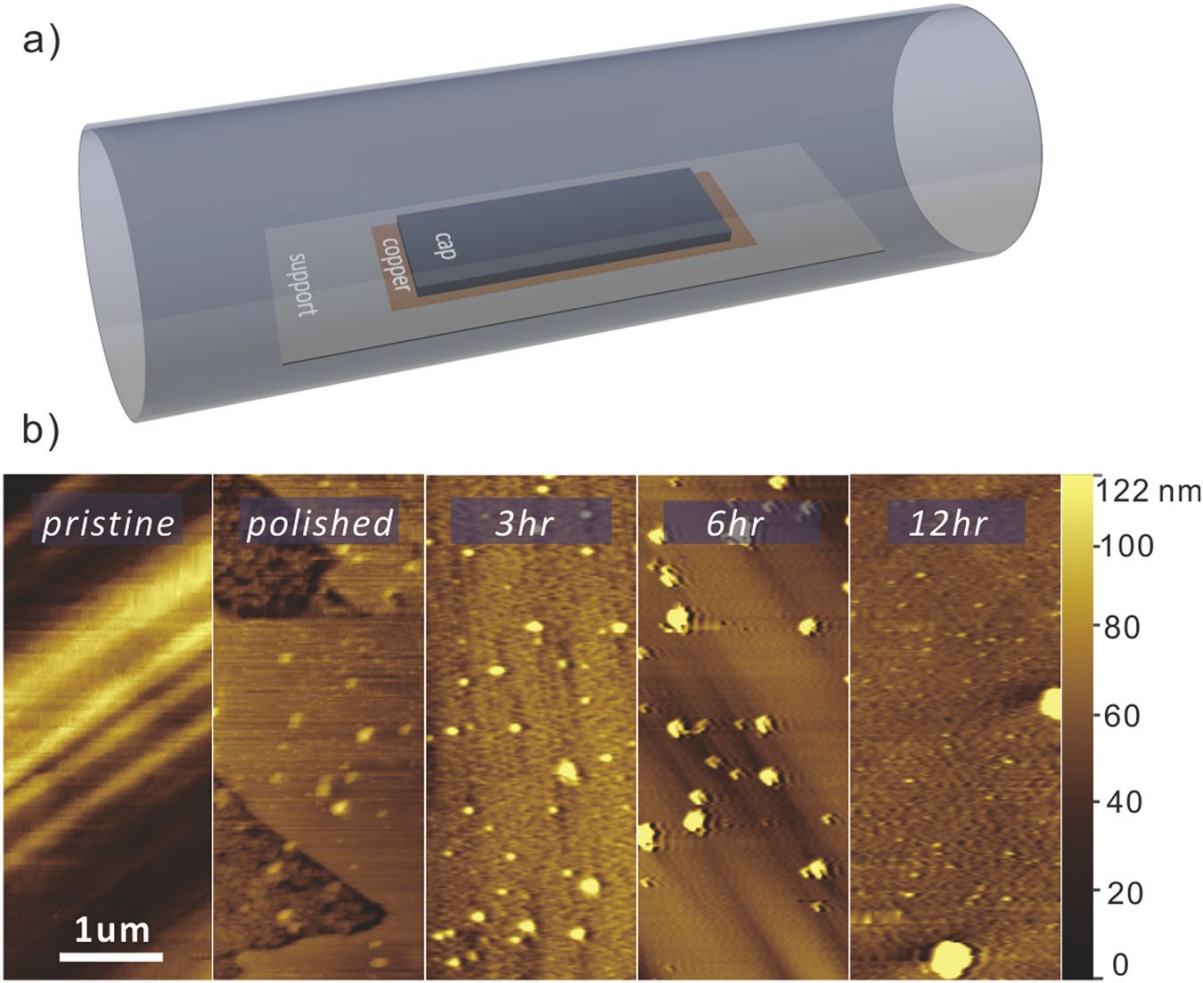
(**a**) Schematics of the capped annealing setup, (**b**) representative AFM images of copper foils as purchased, after electropolishing, and after 3, 6 and 12 hours of annealing at 1020 °C.

We first examine the effect of annealing on the copper foil surface without conducting graphene growth. Figure 1 shows AFM pictures of electropolished copper foil before annealing and after 3, 6, 12 hours of annealing. A clear decrease in the particle concentration can be observed. Several mechanisms could be responsible for this change. First, impurities could diffuse into the bulk due to a difference in concentration at the surface^16^. Furthermore, under elevated temperatures, surface particles of low melting points such as Al will evaporate. Consequently, an increased annealing time helps reduce particle size and numbers. Unfortunately, even after 12 hour annealing surface particles have not completely disappeared. This issue is due to the limited efficiency of the described removal processes. For example, as the concentration of aluminum decreases, the resulting new Cu-Al alloy will exhibit a higher melting point and thus a lower vapour pressure, slowing down the evaporation.

To overcome this issue, we designed a cleaning process that provides an alternative pathway for impurity particles. By placing a material on top of the copper foil, solid diffusion from the copper surface into the cap could occur. This pathway is similar to the gettering method commonly used to remove metal impurities in solar cell-grade silicon^17^ and occurs for every impurity species that exhibits a non-zero mobility in the cap material. The high atomic weight of Cu will furthermore limit the solid diffusion of the catalyst and result in selective absorption of impurities in the cap.

**Figure 2:**
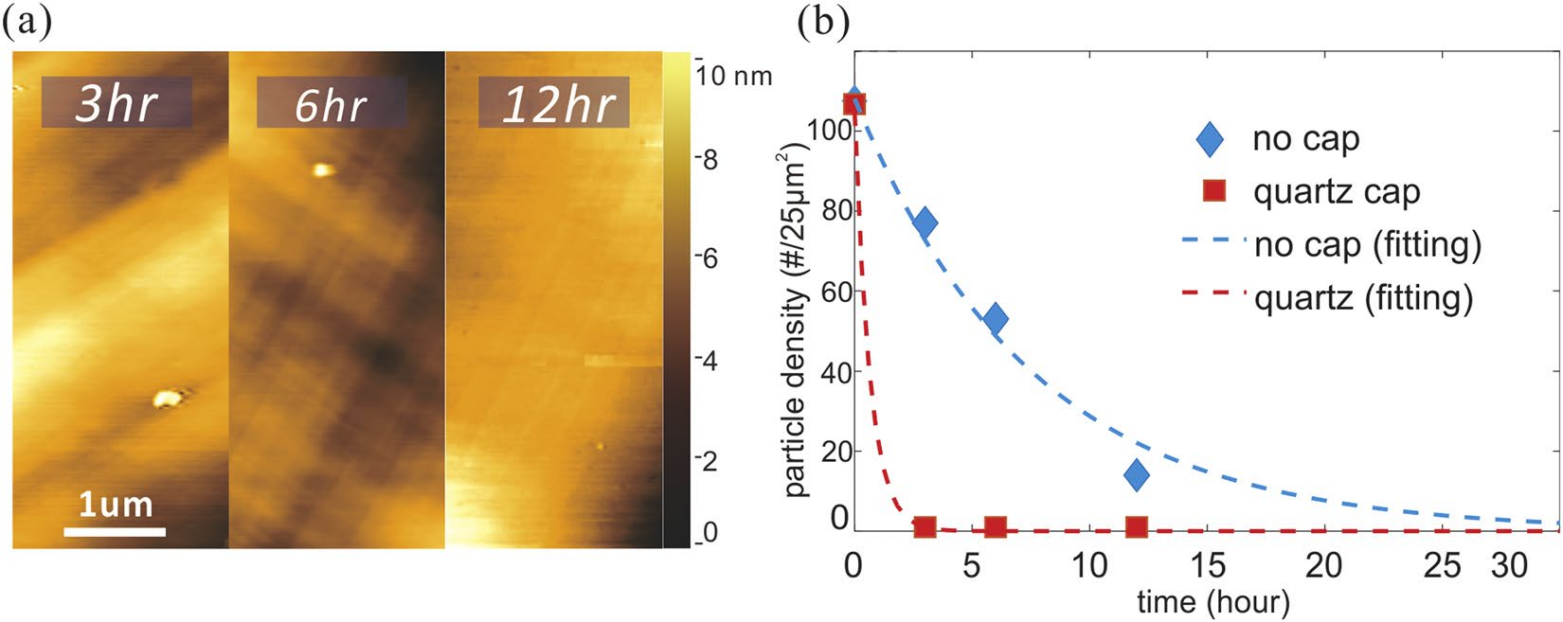
AFM of annealed copper foil with a quartz cap at 1020 °C (**a**) and extracted particle density for annealed copper foils with or without the cap over time (**b**).

We indeed observe that the proposed solid-diffusion-based purification method demonstrated a significant improvement in the particle removal (Fig. 2a). Only 3 hours of annealing were needed to reach ultralow particle densities (1 particle/25µm^2^), which would take normal, uncapped annealing tens of hours to achieve. Fittings of particle densities, obtained from several AFM images for each sample, at different annealing times revealed an exponential decrease whose decay time was 80 times shorter than for uncapped annealing conditions (Fig. 2b).

**Figure 3:**
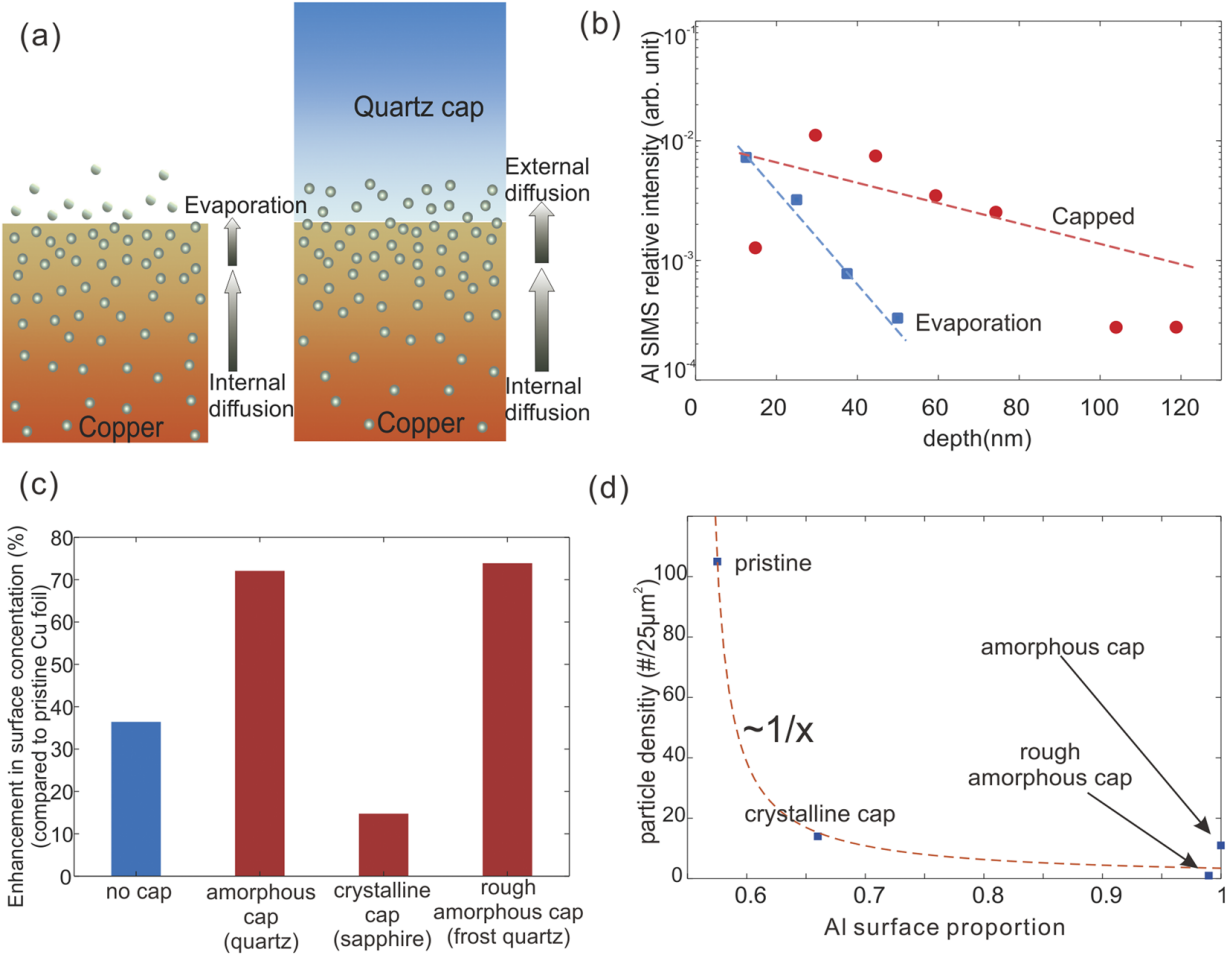
(**a**) Schematic of evaporation and solid diffusion pathways; (**b**) Depth profile of Al in copper foils in non-capped and capped annealing configurations; (**c**) Enhancement in Al surface concentration proportion compared in non-capped and capped annealed copper; (**d**) particle density versus the surface portion of Al in copper foils.

The observed decrease in particle density is due to the increased efficiency in removing impurities from the bulk since solid diffusion into the cap is expected to be faster than evaporation (Fig. 3a). To prove our model, secondary ion mass spectroscopy (SIMS) measurements were carried out on the copper foil after different pretreatment processes. We have found Al, Si, C impurities in the as-received copper foil. Si and C impurities cannot account for the trend in particle density with annealing that was observed in Fig. 1 since their evaporation will not occur at the process conditions. Aluminum, on the other hand, diffuses readily in Cu^18^, aggregates at the surface at annealing temperature, and is thus considered a likely candidate for an active impurity.

We observe a redistribution of Al in the depth profile after annealing compared to the pristine case (Fig. 3b). SIMS depth studies reveal that the proportion of Al in the first 200 nm is increasing for the capped case compared to the evaporation case (Fig. 3b), which confirms that the bulk concentration can be controlled by the efficient removal of impurities from the surface. Our finding is in agreement with a recent study of by Murdock *et al*.^19^, where EDX and XPS measurements detected aluminum appearing on copper foil after annealing. Our model of inter-diffusion was further corroborated by evidence of an increased concentration of Al content was found in the quartz cap (see Fig. S1 in Supplementary Information).

Diffusion can be modified by using different cap materials. A material of denser crystal structure, for example, is expected to exhibit a slower inter-diffusion and a lower efficiency in removing impurities. Indeed, diffusion into sapphire with c-plane (001) cut induced less Al on Cu surface (Fig. 3c) and consequently had higher level of remaining bulk contaminants. Surprisingly, the contact area was found to have a small effect on the diffusion flux and caps with higher surface roughness showed similar enrichment effects. We hypothesize that copper’s high ductility at the annealing temperature produces conformal contact to the cap and highlights the robustness of our approach. Finally, we demonstrate the clear correlation between the observed surface enrichment effect and the concentration of particles that remain on the surface after treatment (Fig. 3d).

It should be noted that beside solid diffusion, capping might cause other effects like strain-induced flattening of copper foil, promoting effect or confinement effect. The promoting effect is precluded from this study by using cap made of inert material (quartz) as opposed to Ni^20^ or graphite^13^. We have previously shown that the gap between substrate and cap alters the fluid dynamics of graphene growth^21^, whereas Chen *et al*.^22^ attributed the confinement to the enhanced redeposition of evaporated Cu, which leads to reduced roughness. While further study needs to be carried out to isolate individual effects, our opinion is that in the case of this study, all the evidences point to the major influence of decreased impurity. In addition to contributing to the surface roughness, impurities with high carbon solubility like Ni^23^ or Al^24^ will be highly likely become graphene nucleation sites.

**Figure 4:**
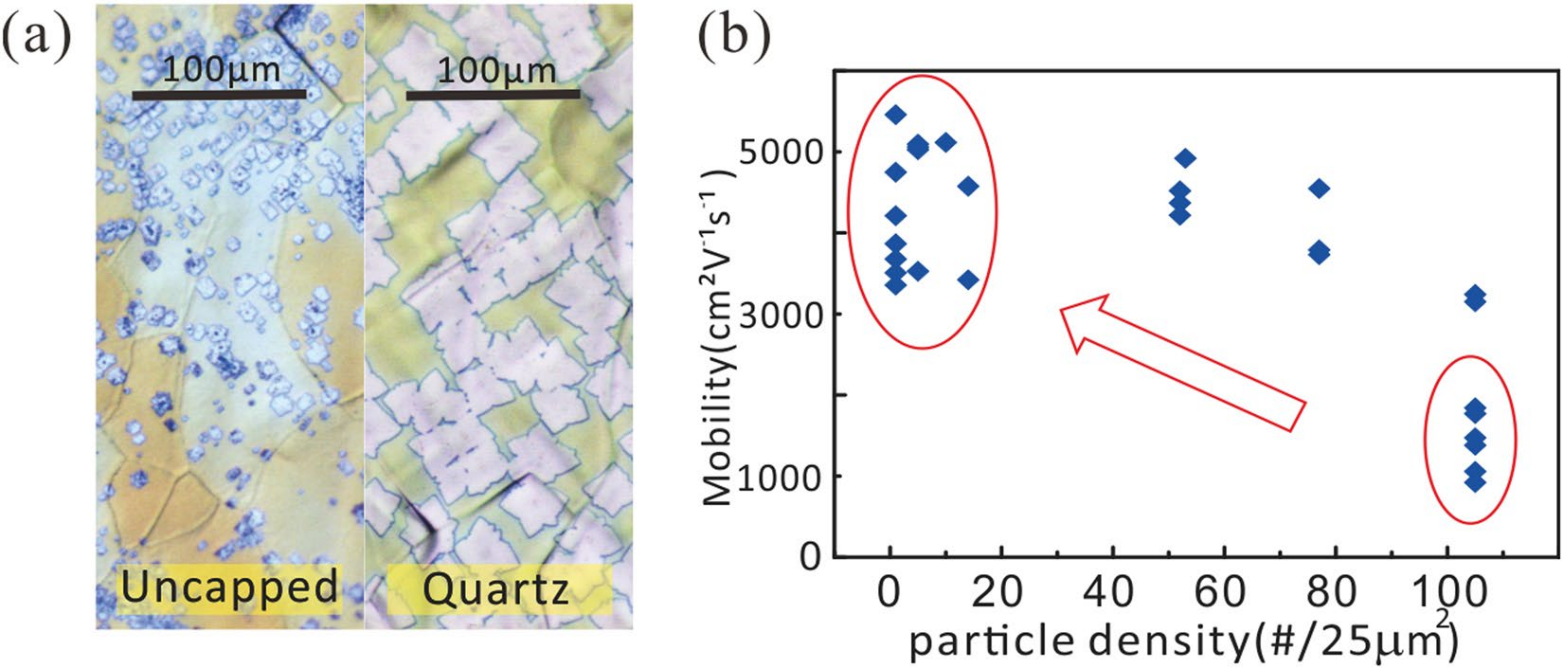
Optical micrographs (**a**) and mobility (**b**) of graphene grown on uncapped and capped copper foils.

Figure 4, Supplementary Figs S2 and S3 depict benefits of copper’s decreased surface-impurity concentration on the graphene quality. Solid-diffusion cleaned copper foils which have fewer surface particles provide fewer seeds for graphene growth. As a result, under the same growth conditions, they are able to grow graphene domains of larger size and higher coverage ratio compared to traditional methods (Fig. 4a). At the same time, the coverage ratio is also improved (Supplementary Fig. S2), due to the enhanced carbon radical diffusion on the smoother surface^25^. A decrease of average I_D_/I_G_ ratio in Raman spectra mapping also confirms the presence of less defects in the capped annealing setup (see Supplementary Fig. S3). Meanwhile, I_2D_/I_G_ ratio is largely invariant between two methods and characteristic of uniformly monolayer graphene (Supplementary Fig. S4). Reduced grain boundary alleviates electron scattering, giving rise to superior electronic properties. Moreover, the reduction of surface-bound particles can decrease the amount of structural defects in the graphene which was found to have a direct effect on the carrier mobility^15^. Consequently, graphene mobility of the capped configuration surpassed that of uncapped one by up to 6 times, which is closely correlated to the improved particle density as established in Fig. 4b. The mobility of up to 5400 cm^2^V^−1^s^−1^ measured at room temperature in large scale is among the highest values for CVD graphene grown under low pressure^26,27^.

## Conclusion

In summary, we have demonstrated an efficient and fast method to control the concentration of surface-bound particles by diffusion from the Cu foil into a solid cap. This control of the density of nucleation seeds allows graphene synthesis with lower grain density and larger size of single crystalline domains. The resulting graphene films exhibit an enhanced mobility and morphology, which highlights the potential of our approach for high quality graphene growth.

## Methods

Copper foil (99.8%, 25 µm, #46365 Alfa Aesar) was electropolished in 85% H_3_PO_4_ for 30 minutes to reduce the roughness and remove impurities on the surface^13^ and then washed in deionized water and dried under a stream of nitrogen. During the annealing process, argon and hydrogen in a 7:2 ratio (350sccm H_2_, 100sccm Ar) were introduced under atmospheric pressure either with or without various caps. The caps used in the study include c-plane sapphire (Al_2_O_3_) (Semiconductor Wafer, Inc.), frosted and polished quartz (XIDE TECHNOLOGY).

Atom force microscopy (Force AFM Genie E7 model) was used to examine copper foil morphology. TOF-SIMS measurement (by a CAMECA IMF 4 f) was carried out using an O_2_^+^ beam of 1 keV to sputter the foil and a Ga^+^ ion beam of 2.5 keV to analyze the secondary ion distribution at the depth up to 1200 nm.

Graphene was grown on the thus treated copper foils with a gas flow of 200 sccm H_2_ and 10sccm CH_4_ at a pressure of 10 Torr for 20 minutes at 1020 °C, followed by a cooling down to room temperature similar to our previous study^28^. Raman measurements were performed in a home-built micro-Raman system using 532 nm laser excitation. FET was fabricated by prepared graphene whence carrier mobility was extracted, with channel length of 250 µm.

## Electronic supplementary material

Supplementary Infomation
