# Supplementary material for: Solid-diffusion-facilitated cleaning of copper foil improves the quality of CVD graphene
Source: Sci Rep. 2019 Jan 22;9:257. doi: 10.1038/s41598-018-36390-4 (PMC6343028; doi:10.1038/s41598-018-36390-4)

Supplementary Information for

Solid-diffusion-facilitated cleaning of copper foil improves the quality of CVD graphene

Dinh-Tuan Nguyen, Wan-Yu Chiang, Yen-Hsun Su, Mario Hofmann ([mario@phys.ntu.edu.tw](mailto:mario@phys.ntu.edu.tw)) and Ya-Ping Hsieh ([yphsieh@gate.sinica.edu.tw](mailto:yphsieh@gate.sinica.edu.tw))

**Supplementary Figure S1**: SIMS depth profile of Al impurity in the quartz cap before and after annealing.


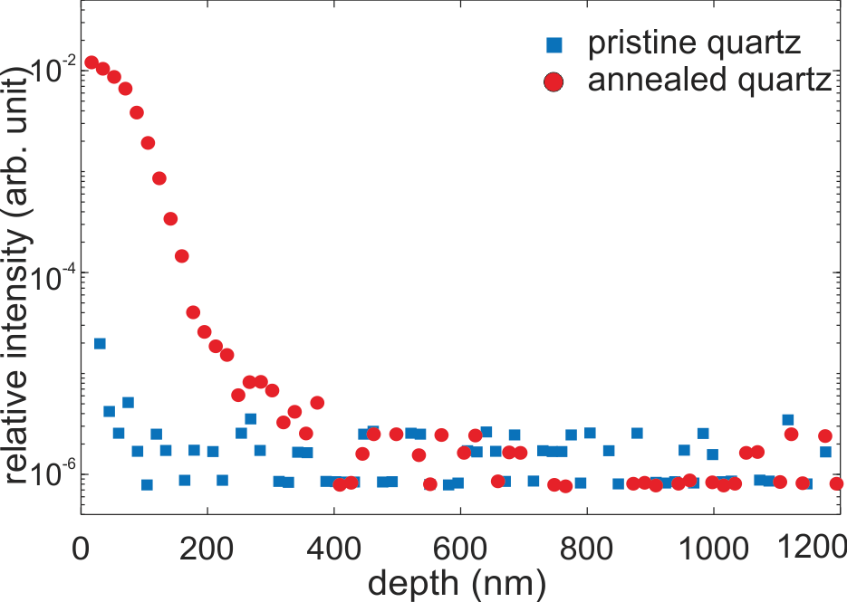


**Supplementary Figure S2:** Comparison of graphene area coverage (%) by uncapped and capped annealing setups (0hr uncapped annealing denotes graphene grown on foil that was electropolished but not annealed).

**Supplementary Figure S3:** Raman mapping of D/G ratio of graphene grown on uncapped and capped annealed foils


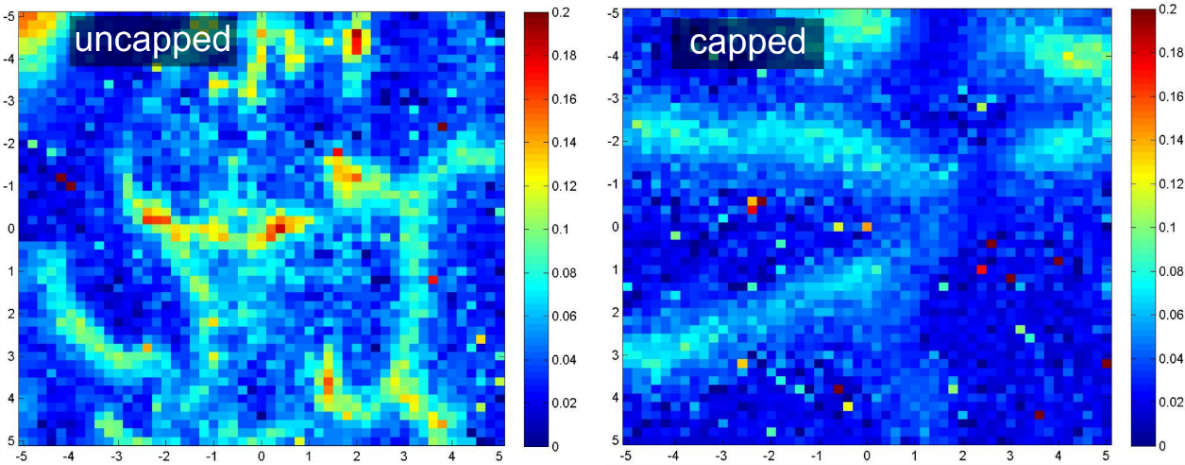


**Supplementary Figure S4:** Raman mapping of 2D/G of graphene grown ratio on not annealed (a), uncapped (b) and capped annealed (c) foils.


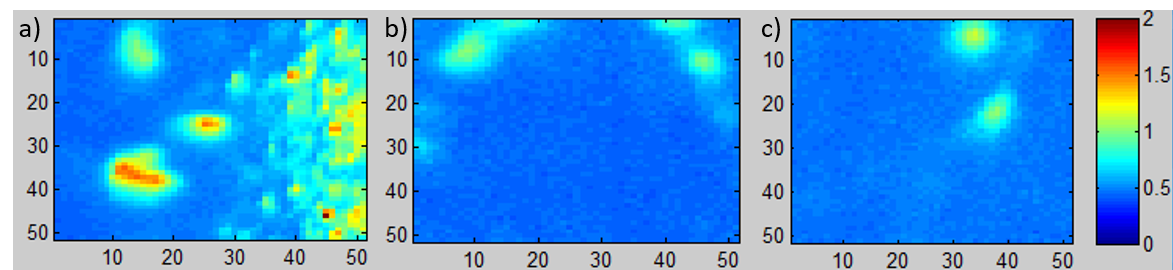


**Supplementary Figure S5**: I-V characteristic (a) and micrograph (b) of FET fabricated from graphene in the capped annealing method.


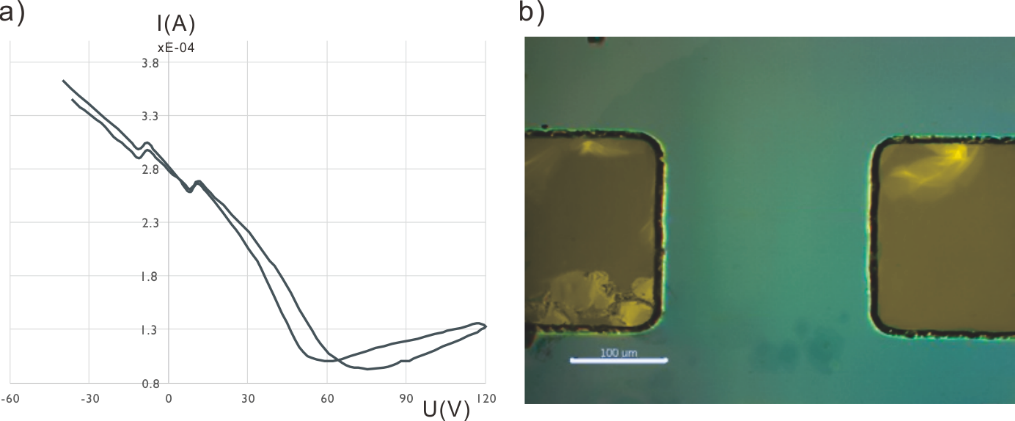

Supplement: Supplementary file 1 — Supplementary Infomation [file 41598_2018_36390_MOESM1_ESM.docx]
